# Low concentrations of tetrasodium EDTA cause significant killing of biofilm-associated Pseudomonas aeruginosa in high-validity models of chronic wound and cystic fibrosis lung infections – but not in a model of endotracheal tube colonization

**DOI:** 10.1099/acmi.0.001155.v4

**Published:** 2026-07-02

**Authors:** Oluwatosin Qawiyy Orababa, Charlotte Cornbill, Ayomikun Kade, Natasha Reddy, Rupika Gulati, Freya Harrison

**Affiliations:** 1 School of Life Sciences, Gibbet Hill Campus, University of Warwick, Coventry, CV47AL, UK; 2 Warwick Medical School, Gibbet Hill Campus, University of Warwick, Coventry, CV47AL, UK; 3 Warwick Manufacturing Group, University of Warwick, Coventry, CV47AL, UK

**Keywords:** biofilms, chronic wounds, cystic fibrosis, endotracheal tube, *Pseudomonas aeruginosa*, tetrasodium EDTA

## Abstract

*Pseudomonas aeruginosa* is a pathogen notorious for its antimicrobial resistance and is currently classified as a high-priority pathogen for which new drugs are needed. Tetrasodium EDTA (tEDTA) is one of the new antimicrobial compounds that have been shown to have good antibacterial and antibiofilm efficacy against *P. aeruginosa*. Due to the diversity and highly drug-tolerant nature of *P. aeruginosa* biofilms in different infection environments, it is important to carry out pre-clinical testing of new antibiofilm agents against this pathogen in media and models that accurately mimic diverse infection environments. In this study, we used different high-validity media and biofilm models that mimic chronic wounds, endotracheal tubes and cystic fibrosis lung infections to assess the efficacy of tEDTA against *P. aeruginosa* biofilms. We report that different infection environments influence the susceptibility of both planktonic and biofilm forms of *P. aeruginosa* to tEDTA. The highest tolerance to tEDTA was observed in the media and biofilm model that mimics the endotracheal tube environment. In conclusion, we show that although different infection environments influence the efficacy of tEDTA against *P. aeruginosa* biofilms, it has good potential for use as an alternative antimicrobial in treating *P. aeruginosa*-associated biofilm infections.

## Data Summary

The authors confirm that all supporting data and protocols have been provided within the article or as supplementary data files (Data S1, available in the online Supplementary Material). Data S1 contains all c.f.u. counts for biofilm eradication experiments in wound, lung and endotracheal tube models.

## Introduction

Antimicrobial resistance (AMR) remains one of the greatest challenges of the twenty-first century with increasing mortality and economic cost. In 2021 alone, there were about 4.71 million deaths associated with bacterial AMR, and this figure has been predicted to rise and reach around 10 million annual deaths by 2050 [1]. Additionally, bacterial AMR accounted for a total hospital cost of US$693 billion and a productivity loss of US$194 billion globally in 2019 [2]. As a result, there is a need for novel classes of antimicrobials and alternative therapeutic options to effectively treat AMR/highly tolerant infections, especially those associated with Gram-negative bacteria [3]. Typical examples of AMR infections are those associated with biofilms. Biofilm-associated infections account for about 65% of all bacterial infections and 80% of all chronic, non-healing microbial infections [4]. These infections, including chronic wounds, ventilator-associated pneumonia (VAP) and cystic fibrosis lung infections, are highly tolerant to antibiotics due to the presence of the biofilm matrix and physiological changes associated with the biofilm state [5].

*Pseudomonas aeruginosa* belongs to the ESKAPE (*Enterococcus faecium*, *Staphylococcus aureus*, *Klebsiella pneumoniae*, *Acinetobacter baumannii*, *P. aeruginosa* and *Enterobacter* spp.) pathogen list of bacteria that are highly resistant to antibiotics [6]. It is also a high-priority pathogen for which new treatments need to be developed and forms highly robust biofilms in sites of infection. *P. aeruginosa* forms biofilms using three main exopolysaccharides (alginate, Pel and Psl), and this may vary depending on the infection site and strain [7,8]. Alginate-encased biofilms are often associated with cystic fibrosis (CF) lung infections, while Pel plays a more significant role in wound infections [7]. Consequently, susceptibility of *P. aeruginosa* biofilms to antibiotics is predicted to be influenced by the infection environment and biofilm matrix type.

Previous research has clearly demonstrated the influence of infection environments on bacterial physiology and their susceptibility to antimicrobials [9,12]. Previous research by our team on in-use antibiotics and candidate novel antibiofilm agents against * P. aeruginosa* has shown discordance between assays conducted in rich medium vs. infection-mimicking medium, and between polystyrene plate-based assays using infection-mimicking medium and high-validity *in vitro* and *ex vivo* infection models [13,14]. It is therefore important to use the right testing models during pre-clinical assessment of antimicrobials with potential to be used in alternative therapies for specific biofilm infection contexts.

Tetrasodium EDTA (tEDTA) has been previously shown to have good antibacterial and antibiofilm activity against various pathogens, including *P. aeruginosa* [15,16]. Liu *et al*. [16] reported the efficacy of 4% tEDTA in eradicating surface-attached biofilms of 12 bacterial pathogens (both Gram-positive and Gram-negative) in central venous access devices. Crowther *et al*. [15] showed that 4% tEDTA caused more than 50% reduction in biofilm biomass of *P. aeruginosa*, and this is further potentiated by the subsequent addition of ciprofloxacin. tEDTA has also been suggested to be a good treatment for wound care [17]. However, previous studies of tEDTA have been conducted in media and biofilm models that do not recapitulate *in vivo* infection environments. Liu *et al*. and Crowther *et al*. grew biofilms attached to polystyrene pegs or microtitre plates, respectively, in rich laboratory growth media.

In this study, we assessed the antibacterial and antibiofilm efficacy of tEDTA against *P. aeruginosa* in three high-validity models of specific biofilm infection contexts, which are in regular use in our lab. These were an *in vitro* model of soft-tissue chronic wound biofilm [18], a porcine *ex vivo* model of biofilm in the lungs of people with cystic fibrosis [19] and an *in vitro* model of biofilm in the endotracheal tubes (ETTs) used to connect hospital patients to ventilators [20]. *P. aeruginosa* is one of the most predominant pathogens in chronic wounds, CF lung and VAP infections due to its ability to form robust biofilms in these environments [21]. Hence, identifying effective treatment for this pathogen in relevant models that recapitulate these infection environments is essential towards reducing *P. aeruginosa* morbidity and mortality. Here, we used a well-characterized laboratory strain of * P. aeruginosa*: PA14, which can produce the pel and alginate polysaccharides, as well as proteinaceous and nucleic acid matrix components. We showed that although tEDTA has good antibiofilm efficacy against *P. aeruginosa* in the different models, the level of efficacy varies by growth environment. Biofilms grown in the ETT model were notably harder to eradicate with tEDTA than biofilms grown in the other two models.

## Methods

### MIC assay

The broth microdilution method was used to determine the MIC of tEDTA (Sigma-Aldrich, UK) as recommended by the European Committee on Antimicrobial Susceptibility Testing. Briefly, *P. aeruginosa* PA14 was streaked on Luria–Bertani (LB) agar (Sigma-Aldrich) and incubated at 37 °C for 18–24 h to produce distinct colonies. Twice the maximum concentration of tEDTA to be used was prepared in the medium of interest [caMHB (Sigma-Aldrich), SWF (50% v/v FBS (Gibco) and peptone water (Sigma-Aldrich)) [18], SCFM1 [22] and SVAM [20,23]] and dispensed (100 µl) in the first column of a Corning Costar CLS9018 (Corning Inc., USA) 96-well plate. Fifty microlitres of the medium were then dispensed into each of the other wells, after which a twofold serial dilution was carried out. Bacterial suspensions were prepared by touching three to four distinct colonies with a sterile cotton swab and dispersing them in PBS. This was then standardized to a 0.5 McFarland standard with a spectrophotometer (OD600nm=0.08–0.10). The standardized bacterial suspension (50 µl) was then inoculated into triplicate wells of each concentration except for the sterile control well (without tEDTA or bacteria). Growth control wells with bacteria (without tEDTA) and with no bacteria were also set up. The 96-well plates (Corning Inc., USA) were sealed with Parafilm™ (Bemis) and incubated at 37 °C for 18–24 h. The lowest concentration well with no observable growth was taken as the MIC.

### Biofilm eradication assay in the synthetic collagen wound biofilm model

A synthetic soft-tissue wound model was used as previously described by Werthén *et al*. [18]. Synthetic wounds were prepared using a mixture of 20% rat tail collagen type 1 (10 µg ml^−1^) (Corning), 60% SWF, 10% of 0.1% acetic acid (Sigma-Aldrich) and 10% of 10 mM sodium hydroxide (Sigma-Aldrich). The mixture was placed on ice and mixed slowly to avoid bubbles. Following this, 200 µl of the mixture was added to wells in a 48-well plate (Greiner Bio-One – 677 180). Synthetic wounds were sterilized under short-wave UV light for 10 min before being incubated at 37 °C for 1 h to allow the collagen matrix to polymerize. A few colonies of *P. aeruginosa* PA14 from an overnight LB plate were inoculated into 5 ml SWF and incubated with shaking at 37 °C for 6 h. This culture was diluted in SWF to obtain an OD_600_ of 0.08–0.1. Fifty microlitres of standardized culture were added to the collagen wound bed and incubated at 37 °C for 24 h. After 24 h, 100 µl of tEDTA at 0.5% or 1%, or sterile water (untreated control), was added to the synthetic wounds and incubated at 37 °C for 24 h. Following this, 300 µl of collagenase type 1 (0.5 mg ml^−1^) was added to each wound and incubated for 1 h at 37 °C to dissolve the collagen matrix. Each well was then mixed thoroughly by pipetting, serially diluted and plated on LB agar plates that were incubated overnight at 37 °C. Bacterial colony counts were used to calculate c.f.u. per wound.

### Biofilm eradication assay in the *ex vivo* pig lung model

The *ex vivo* pig lung (EVPL) model has previously been described [19,24, 25]. Briefly, porcine lungs were obtained from a local butcher (Taylors Butchers, Earlsdon). The bronchioles were dissected and washed in 1 : 1 RPMI:DMEM with 20 µg ml^−1^ ampicillin. The bronchioles were dissected into 5 mm strips and washed again in 1 : 1 RPMI:DMEM. Following this, they were dissected into 5 mm squares and washed for a third time in 1 : 1 RPMI:DMEM and then in synthetic cystic fibrosis sputum media (SCFM1) prepared as described previously [22], but without glucose. Following these washes, bronchiole tissue was sterilized under short-wave UV light in SCFM1 for 10 min. The tissue sections were placed individually into the wells of a flat, clear 24-well plate (Corning) containing 400 µl of 0.8% w/v agarose in SCFM1. Each piece of tissue was inoculated with PA14 from an overnight LB plate by using a sterile 29G hypodermic needle lightly touching a colony, then lightly piercing the tissue ~10 times. After inoculation, 500 µl of SCFM1 was added to each well, and the plate was sealed with a Breathe-Easier^®^ membrane (Diversified Biotech) and incubated at 37 °C for 48 h. Following incubation, bronchiole tissue with associated biofilm was briefly washed in 500 µl of PBS to remove loosely adhering planktonic bacteria and placed into a fresh 48-well plate, containing 1%, 2% or 3% tEDTA in SCFM1, or SCFM1 only (untreated control). The plate was sealed with a Breathe-Easier^®^ membrane and incubated at 37 °C for 24 h. Following treatment, the tissue and associated biofilm were briefly washed in 500 µl of PBS and placed into sterile homogenization tubes each containing 18 2.38 mm metal beads (FisherBrand) and 1 ml PBS. The tissue was homogenized in a FastPrep-24 5 G (MP Biomedicals) for 40 s at 4 ms−1. The homogenate was then serially diluted and plated onto LB agar, which was incubated at 37 °C overnight to determine the c.f.u. per lung piece.

### Biofilm eradication assay in the *in vitro* ETT model

The *in vitro* endotracheal tube (IVETT) model has been previously described [20]. ETTs (siliconized PVC, cuffed, 8 mm; IMS Euro) were prepared by cutting a 1 cm ring from the tube and chopping this radially into six equal parts under aseptic conditions. Cut sections were sterilized under short-wave UV light for 10 min and covered with FBS (Gibco) in a Petri dish. This was sealed with Parafilm™ (Bemis) and left at 4 °C overnight for serum coating. Serum-coated ETT sections were added individually to the wells of 24-well plates (Corning Costar) using sterile forceps and allowed to dry for 30 min. An overnight culture of *P. aeruginosa* PA14 in LB agar was standardized in SVAM [23] to 0.1 OD_600_, and 0.5 ml was added to each tube section. The plates were then incubated at 37 °C, 5% CO_2_ for 24 h, and the biofilm-coated tubes were transferred into fresh SVAM medium for another 24 h. Tubes were transferred to fresh SVAM containing tEDTA at a concentration of 2%, 3% or 4%, or to fresh SVAM only (no treatment control) and incubated at 37 °C, 5% CO_2_ for 24 h. Subsequently, the tubes were transferred to 500 ml PBS, sonicated at 50 Hz (Grant XUBA1 sonicating water bath) for 15 min and agitated further to ensure complete removal of biofilm from the ETT. Serial dilution was performed before plating out on LB agar to determine the c.f.u. per ETT section.

### Statistical analysis

All data visualization and statistical analysis were done with the *ggplot2* and *multcomp* packages in R (version 4.5.2).

## Results and discussion

### tEDTA has good antibacterial efficacy against planktonic *P. aeruginosa* in standard rich medium and host-mimicking media

Environmental cues in various infection environments play crucial roles in determining bacterial susceptibility to antibiotics [9,14]. To determine the impact of growth medium on the susceptibility of *P. aeruginosa* to tEDTA, we first assessed its MIC against planktonically grown *P. aeruginosa* PA14 using the broth microdilution assay in both standard laboratory media (caMHB) and infection-mimicking media. The infection-mimicking media were simulated wound fluid (SWF [18]), synthetic cystic fibrosis sputum media (SCFM1 [22]) and synthetic ventilated airway mucus (SVAM [23]), – these media can be used to make high-validity biofilm platforms that mimic the environments of soft-tissue wounds, CF airways and ETTs, respectively.

tEDTA inhibited the growth of *P. aeruginosa* PA14 in all the media tested with MICs ranging from 0.25% to 1% (Table 1). This was consistent across replicates. The highest MIC was seen with PA14 in the SVAM; this strain had a fourfold variation in MIC across the media tested. This evidenced the role of growth environment in the susceptibility of this pathogen to tEDTA, even though the variation between media tested was relatively small. The antibacterial efficacy of tEDTA against *P. aeruginosa* PA14 in this study is in line with previous studies that have reported that this compound has good efficacy against *P. aeruginosa* [15,26]. The MIC (0.25%) reported in this study in both the standard lab medium (caMHB) and SWF is also similar to the MIC reported by Sivaranjani *et al*. [26]. However, we report a slightly higher (0.5–1%) MIC in SCFM and SVAM against *P. aeruginosa* PA14. This higher MIC seen in SCFM and SVAM might be associated with the presence of potassium compounds (e.g. KCl and K_2_SO_4_) in both media [19,20]. Potassium is a more reactive metal than sodium and hence can displace sodium from tEDTA, thereby reducing the efficacy of tEDTA against *P. aeruginosa* in these two media.

**Table 1.** MICs of tEDTA in different media

	caMHB	SWF	SCFM	SVAM
PA14 MIC (% w/v)	0.25	0.25	0.5	1

### tEDTA causes significant killing of mature *P. aeruginosa* biofilm, in a soft tissue chronic wound model

**Fig. 1.**
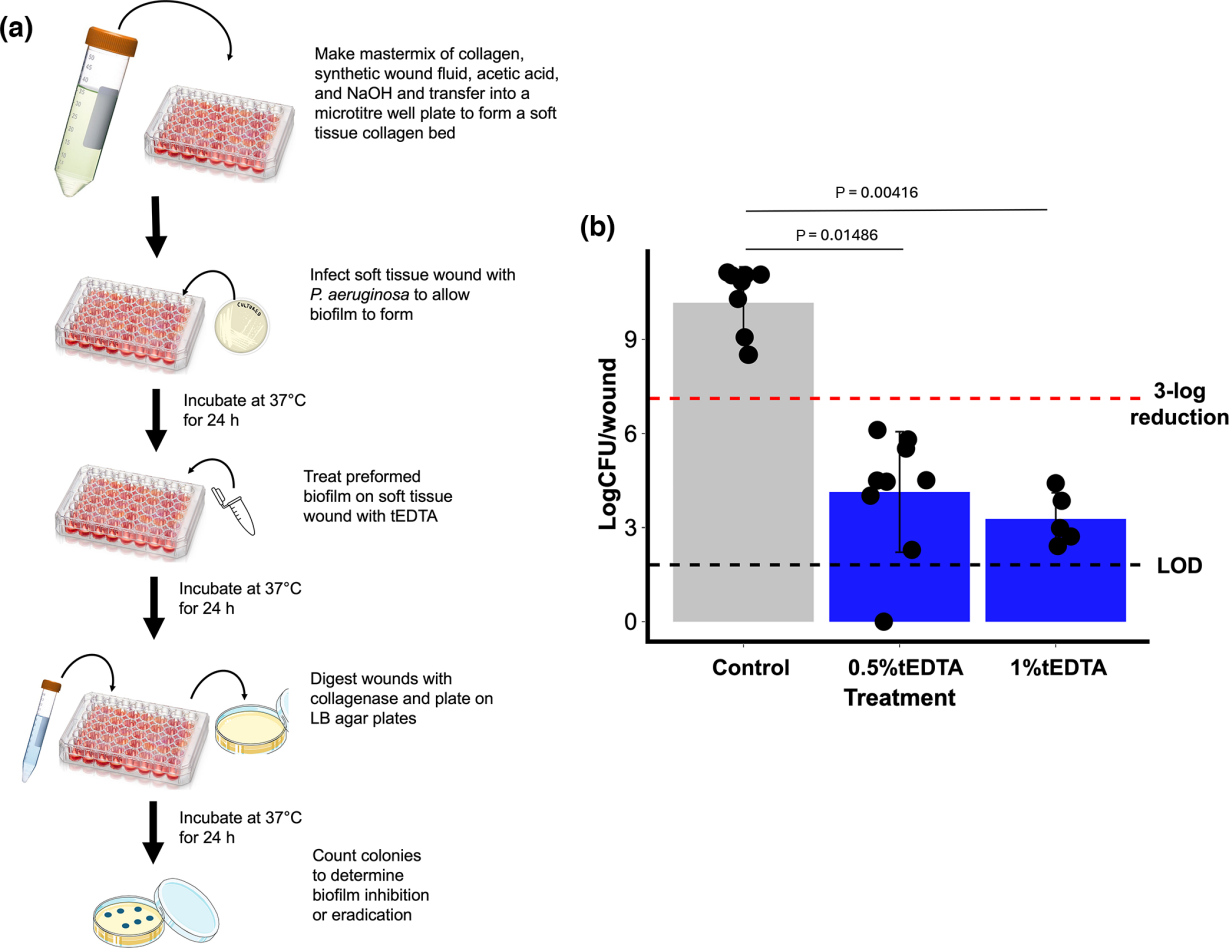
Assays for prevention and eradication of *P. aeruginosa* biofilm in a soft tissue chronic wound model. (a) Flow diagram showing how the soft tissue wound biofilm model was used for assessing biofilm inhibition and eradication. (**b**) A soft tissue wound was infected with *P. aeruginosa* PA14 and incubated for 24 h to allow biofilms to form. The preformed biofilms were treated with different concentrations (0.5% and 1%) of tEDTA and incubated at 37 °C for another 24 h. Bacteria were recovered by enzymatic digestion of the collagen and c.f.u. counted to determine viable cell numbers. The red dashed line indicates a 3-log_10_ bacterial reduction; the black line indicates the limit of detection (LOD) for c.f.u. by plating. Error bars are mean±sd of three independent experiments and three technical replicates for each (*N*=3, *n*=3). Results were analysed with Tukey’s pairwise comparison test after ANOVA. There was a significant effect of treatment [F(2,20)=8.164, *P*<0.01].

*P. aeruginos*a is one of the major bacterial pathogens in chronic wound infections and is associated with treatment difficulty [27]. We assessed the ability of tEDTA to inhibit biofilm formation or to eradicate an established biofilm of *P. aeruginosa* PA14 strain in a soft tissue chronic wound model (Fig. 1a) [18]. Werthén *et al*. [18] previously showed that the structure of *P. aeruginosa* biofilm formed in this *in vitro* model is similar to its structure *in vivo*. We also allowed biofilms to grow in the model for 24 h, before adding the treatment for a further 24 h. Both 0.5% tEDTA and 1% tEDTA caused a more than 3-log_10_ reduction in biofilm population (Fig. 1b and Data S1). Interestingly, there was no significant difference in activity between the 0.5% and 1% tEDTA (*P*>0.05). This is the first report of the biofilm eradication efficacy of tEDTA in this chronic wound model. Crowther *et al*. [15] showed that treatment of *P. aeruginosa* biofilm with 4% tEDTA resulted in a 3-log_10_ reduction in biofilm population using the polystyrene microtitre plate platform and rich growth medium. However, in our study, we observed a 3-log_10_ reduction with a much lower tEDTA concentration than that used by Crowther *et al*. [15] – indicating lower tolerance of biofilm to tEDTA in the wound model. Also, the variations in concentration might be linked to differences in *P. aeruginosa* strain used. Crowther *et al*. [15] used PAO1, while we used the PA14 strain in this study. These two strains produce different biofilm-associated exopolysaccharides [7,8].

### tEDTA can completely eradicate mature *P. aeruginosa* biofilm in a CF lung model

**Fig. 2.**
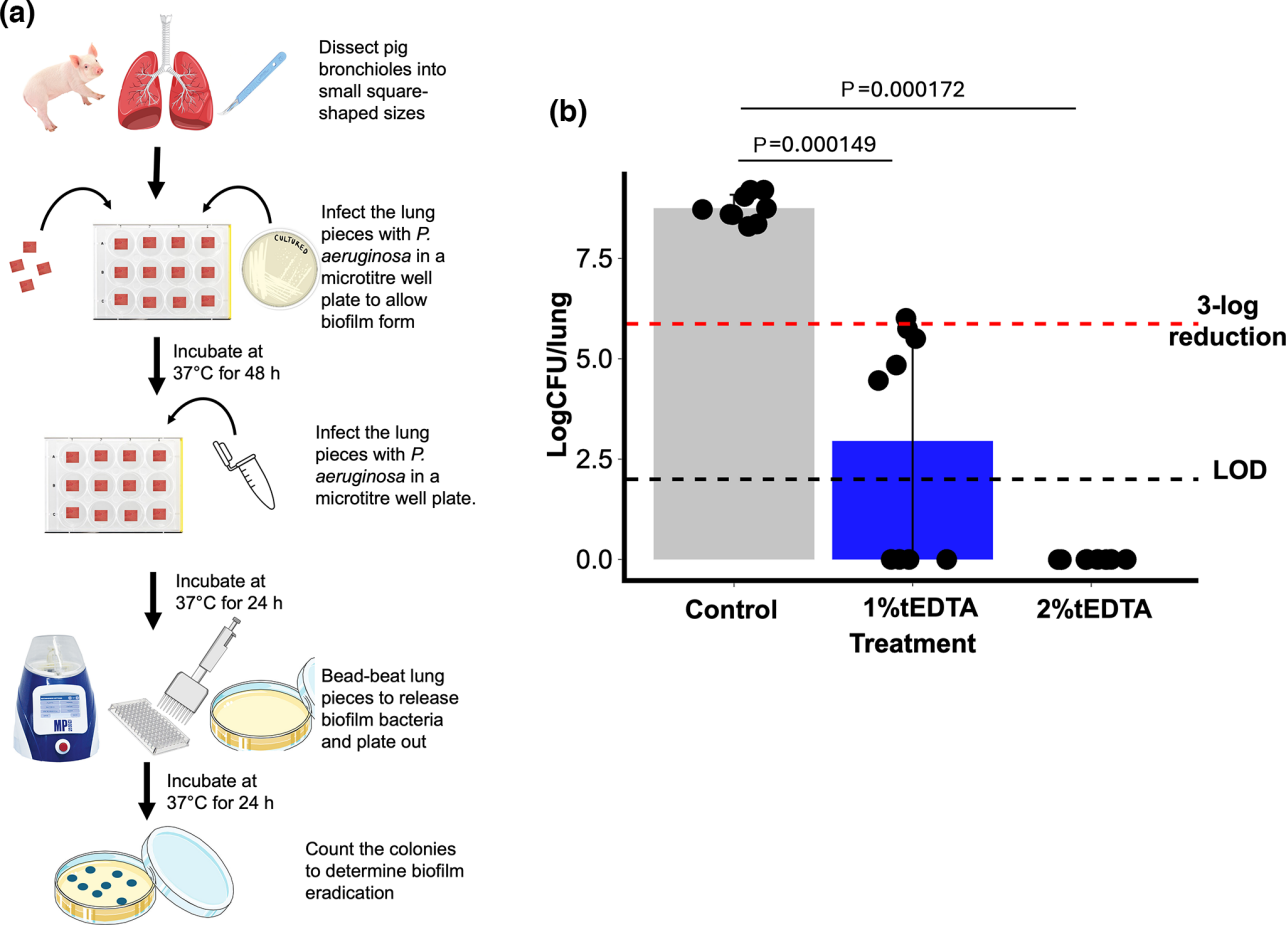
Assay for eradication of *P. aeruginosa* biofilm in an *ex vivo* cystic fibrosis lung model. (a) Flow diagram showing how the EVPL model of CF infection was used to assess biofilm eradication. (**b**) Bronchioles from pig lungs were dissected into small pieces and infected with *P. aeruginosa* PA14. The infected lung was incubated at 37 °C for 48 h to allow biofilms to form. After 48 h, the lungs were treated with different concentrations (1% and 2%) of tEDTA and incubated for a further 24 h before bacteria were recovered from the tissue-associated biofilm by bead beating and c.f.u. counted to determine viable cell numbers. The red dashed line indicates a 3-log_10_ bacterial reduction; the black line indicates the LOD for c.f.u. by plating. Error bars are mean±sd of three independent experiments and three technical replicates for each (*N*=3, *n*=3). Results were analysed with Tukey’s pairwise comparison test after ANOVA. There was a significant effect of treatment [F(2,24)=15.69, *P*<0.001].

Chronic *P. aeruginosa* lung infection is prevalent among adults with CF and is responsible for a significant decrease in health and life expectancy [28,29]. We assessed the ability of tEDTA to eradicate preformed biofilm of *P. aeruginosa* in a CF lung model (Fig. 2a) which has been shown to induce high-level tolerance to antibiotics [14,19, 24]. tEDTA caused more than a 3-log_10_ reduction in biofilm population of *P. aeruginosa* PA14 when used at 1% in this model (Fig. 2b and Data S1). Interestingly, 2% tEDTA completely eradicated *P. aeruginosa* biofilm in this model (Fig. 2b). However, statistical analysis revealed no significant difference in the biofilm eradication activity of 1% tEDTA and 2% tEDTA (*P*>0.05) in the lung model. To our knowledge, no previous study has reported the effect of tEDTA on CF lung pathogens. The good biofilm eradication efficacy we see with tEDTA against *P. aeruginosa* in this CF lung model indicates its potential for development into a nebulized treatment or a sinus wash that could manage CF lung infections resulting from *P. aeruginosa*.

### Biofilm eradication of *P. aeruginosa* with tEDTA is more difficult in an ETT model

**Fig. 3.**
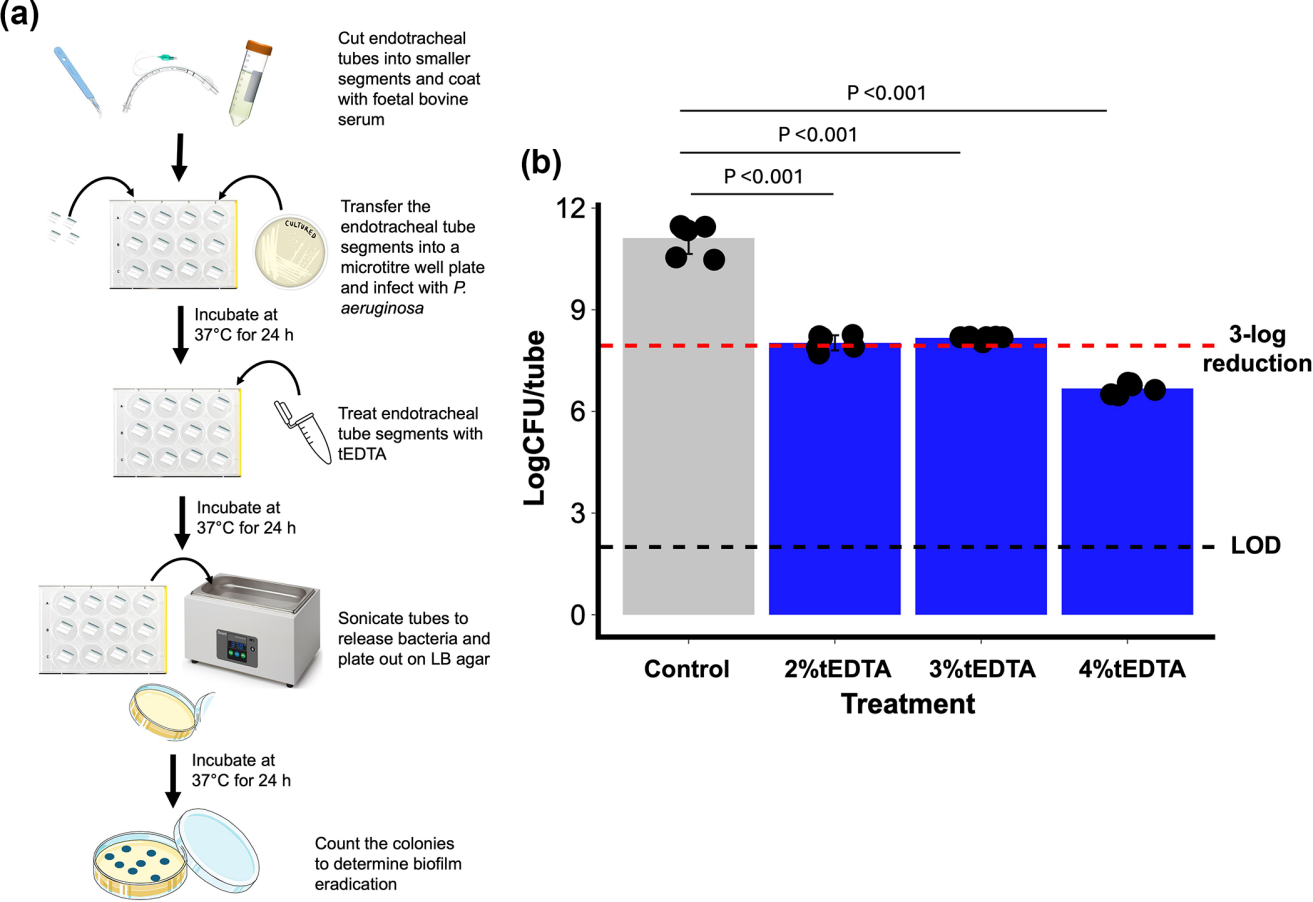
Assay for eradication of *P. aeruginosa* biofilm with tEDTA in an IVETT biofilm model. (a) Flow diagram showing how the IVETT model was used to assess biofilm eradication. (**b**) Segments of ETTs were coated with FBS, infected with *P. aeruginosa* PA14 and incubated for 48 h in SVAM. Tubes were then treated with specific concentrations (2%, 3% and 4%) of tEDTA for 24 h. After this, bacteria were recovered from the ETT-associated biofilm by sonication and c.f.u. enumerated. The red dashed line indicates a 3-log_10_ bacterial reduction. Error bars are mean±sd of three independent experiments and two technical replicates for each (*N*=3, *n*=2). Results were analysed with Tukey’s pairwise comparison test after ANOVA. There was a significant effect of treatment [F(3,20)=14.02, *P*<0.001].

VAP is one of the most common nosocomial infections in critically ill patients, with *P. aeruginosa* being a common pathogen in VAP [30]. VAP is ultimately a consequence of biofilm formation on the ETT used to connect the patient to the ventilator: fragments of the ETT-associated biofilm colonize the lungs, leading to pneumonia [31,33]. Using an IVETT model that was recently developed in our lab [20], we assessed the ability of tEDTA (2%, 3% and 4%) to eradicate mature *P. aeruginosa* biofilm on an abiotic surface (Fig. 3a). Among the tested concentrations, only 4% tEDTA yielded more than a 3-log_10_ reduction in viable bacteria (Fig. 3b and Data S1). This was higher than the concentration required to achieve at least 3-log_10_ biofilm killing in the wound (0.5%) or EVPL (1%) models. However, this concentration (4%) is deemed safe and is currently used in the clinic to prevent bloodstream infections associated with central venous catheters [34]. The concentration of tEDTA required to achieve a 3-log_10_ reduction in biofilm population in the IVETT is the same as the concentration required by Crowther *et al*. [15] to cause a comparable killing of biofilms grown in polystyrene microtitre plates. Although no study has previously checked the antibiofilm efficacy of tEDTA in the IVETT model, previous work has explored the impact of tEDTA on other infections associated with medical device biofilms. For example, Robinson *et al*. [34] showed that 4% tEDTA prevents central-venous catheter-associated bloodstream infections in paediatric haemodialysis patients. Moore *et al*. [35] also showed that about 1% tEDTA reduced the risk of catheter blockade with biofilms in an *in vitro* catheter-associated urinary tract infection model. Similarly, Hirsch *et al.* [36] showed that 4% tEDTA reduced the risk of composite catheter complications in paediatric patients. Additionally, *P. aeruginosa* PA14 grew to its highest average population (in the control) in the ETT model compared to the other two models, and this might have also contributed to its high tolerance in this model compared to the wound and CF model.

## Conclusions

In this study, we showed that different biofilm infection environments are likely to influence the susceptibility of *P. aeruginosa* biofilm to tEDTA. Nonetheless, this non-antibiotic antimicrobial has the potential to be used to treat some *P. aeruginosa* biofilm infections. We have also shown that the antibiofilm efficacy of at least 3-log_10_ is achieved at concentrations below the already clinically approved concentration (4%) of tEDTA in catheter-associated bloodstream infections. Future studies should focus on testing tEDTA on a wider panel of laboratory and clinical strains of *P. aeruginosa*, as well as a wider panel of Gram-negative pathogens. Additionally, future studies should assess the suitability of tEDTA for incorporation into wound care products, or into solutions that could be nebulized into the CF lung, or used as washes for sinus decontamination in CF. We did not find promising activity when we tried to eradicate ETT biofilms of *P. aeruginosa* with tEDTA at concentrations of up to 4%. Future work could also usefully explore the ability of tEDTA to prevent or slow biofilm colonization of ETTs; this may be achievable and could potentially translate into development of coated ETTs that are functionalized with tEDTA. Similarly, experiments assessing the ability of lower concentrations of tEDTA may indicate promise for the prevention of *P. aeruginosa* colonization of wound infections or CF lungs. Parallel safety/toxicity testing with human cell lines or tissues should ideally be conducted. The mechanism by which EDTA affects bacteria/biofilm is thought to be by chelating cations (Ca^2+^ and Mg^2+^) on the membrane, which likely disrupts bacterial membrane potential [37]. Future studies should also identify the specific mechanism of action of tEDTA against *P. aeruginosa*. In conclusion, our work shows the potential of tEDTA as an antimicrobial agent for different *P. aeruginosa*-associated biofilm infections and how different infection environments can influence its antibiofilm efficacy against *P. aeruginosa*.

## Supplementary material

10.1099/acmi.0.001155.v4 Supplementary Material 1.

## Abbreviations

AMR: antimicrobial resistance
CF: cystic fibrosis
ETTs: endotracheal tubes
EVPL: *ex vivo* pig lung
IVETT: *in vitro* endotracheal tube
LB: Luria–Bertani
LOD: limit of detection
SVAM: synthetic ventilated airway mucus
tEDTA: tetrasodium EDTA
VAP: ventilator-associated pneumonia
